# Primary hydatid cyst in the adductor magnus muscle

**DOI:** 10.1259/bjrcr.20200019

**Published:** 2020-03-11

**Authors:** Meltem Özdemir, Rasime Pelin Kavak, Nezih Kavak, Noyan Can Akdur

**Affiliations:** 1 Department of Radiology, Dışkapı Yıldırım Beyazıt Training and Research Hospital, University of Health Sciences, Ankara, Turkey; 2 Department of Emergency Medicine, Dışkapı Yıldırım Beyazıt Training and Research Hospital, University of Health Sciences, Ankara, Turkey; 3 Department of Pathology, Ankara Keçiören Training and Research Hospital, Ankara, Turkey

## Abstract

Human hydatid cyst is a zoonotic disease caused by the larvae of the *Echinococcus* species, most commonly the *Echinococcus granulosus*. Although hydatid cyst can cause disease almost anywhere in the human body, it most commonly affects the liver and lungs. Primary musculoskeletal hydatid involvement is a very rare occurrence even in endemic regions. Musculoskeletal hydatid disease shows no pathognomonic clinical signs and symptoms. And the contribution of serology to the diagnosis is negligible due to the high rate of false-negative results. Therefore, radiological imaging studies have a critical role in the diagnosis of the disease. To the best of our knowledge, there are only a few case reports of primary hydatid involvement of the adductor magnus muscle in the current literature. Here we present a 55-year-old female patient with primary hydatid cyst in the adductor magnus muscle and discuss the case in terms of imaging.

## Case presentation

A 55-year-old female presented with painless swelling in the right thigh over the last few weeks. The patient had no history of fever, tremor, pruritus, or trauma to the thigh. On physical examination, there was a non-tender mass on the medial side of the right upper thigh the borders of which were not clearly distinguishable and did not show any signs of inflammation on the skin.

## Imaging findings

**Figure 1.**
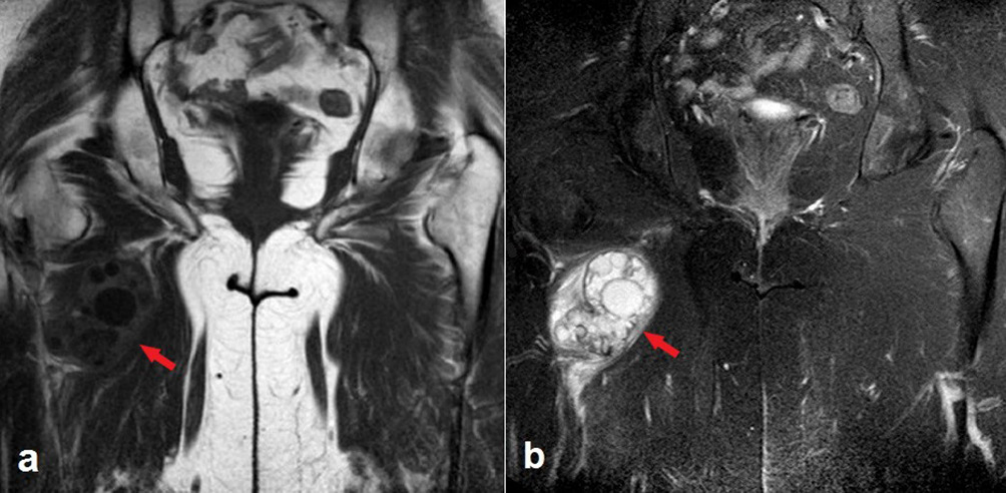
Coronal *T*_1_- (a) and fat-suppressed *T*_2_ weighted (b) magnetic resonance images show a hydatic cyst lesion including multiple daughter cysts within the right adductor magnus muscle (arrows).

**Figure 2.**
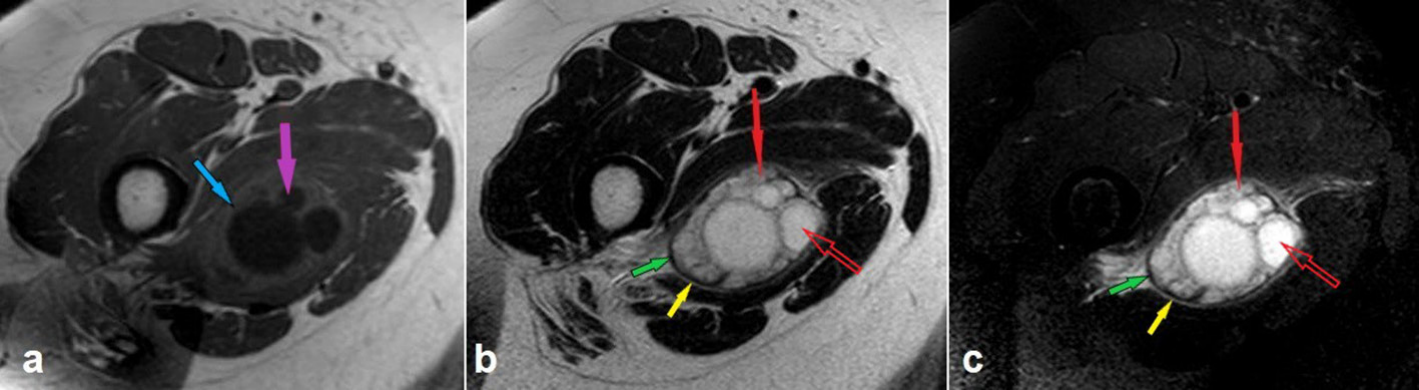
Axial *T*_1_- (a), *T*_2_- (b), and fat-suppressed *T*_2_ weighted (c) magnetic resonance images. The daughter cysts are hypointense (purple arrow) and the mother cyst is iso-hyperintense (blue arrow) as compared with the adjacent muscle tissue in *T*_1_ weighted image. In *T*_2_ weighted images, both the mother (solid red arrows) and daughter (open red arrows) cysts show high signal intensity. And *T*_2_ weighted images nicely delineate the hypointense ectocyst (green arrows) and hyperintense pericyst (yellow arrows) surrounding the lesion.

**Figure 3.**
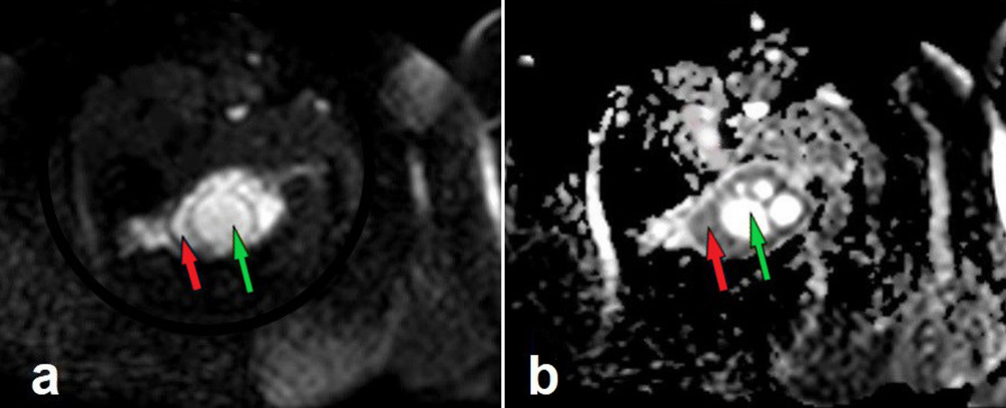
DW MRI (a) and ADC mapping (b). While the daughter cysts appear hyperintense in both DWI and ADC mapping (yellow arrows), there is a prominent restriction of diffusion in the remaining areas of the lesion (blue arrows). ADC, apparentdiffusion coefficient; DWI,diffusion-weighted imaging.

**Figure 4.**
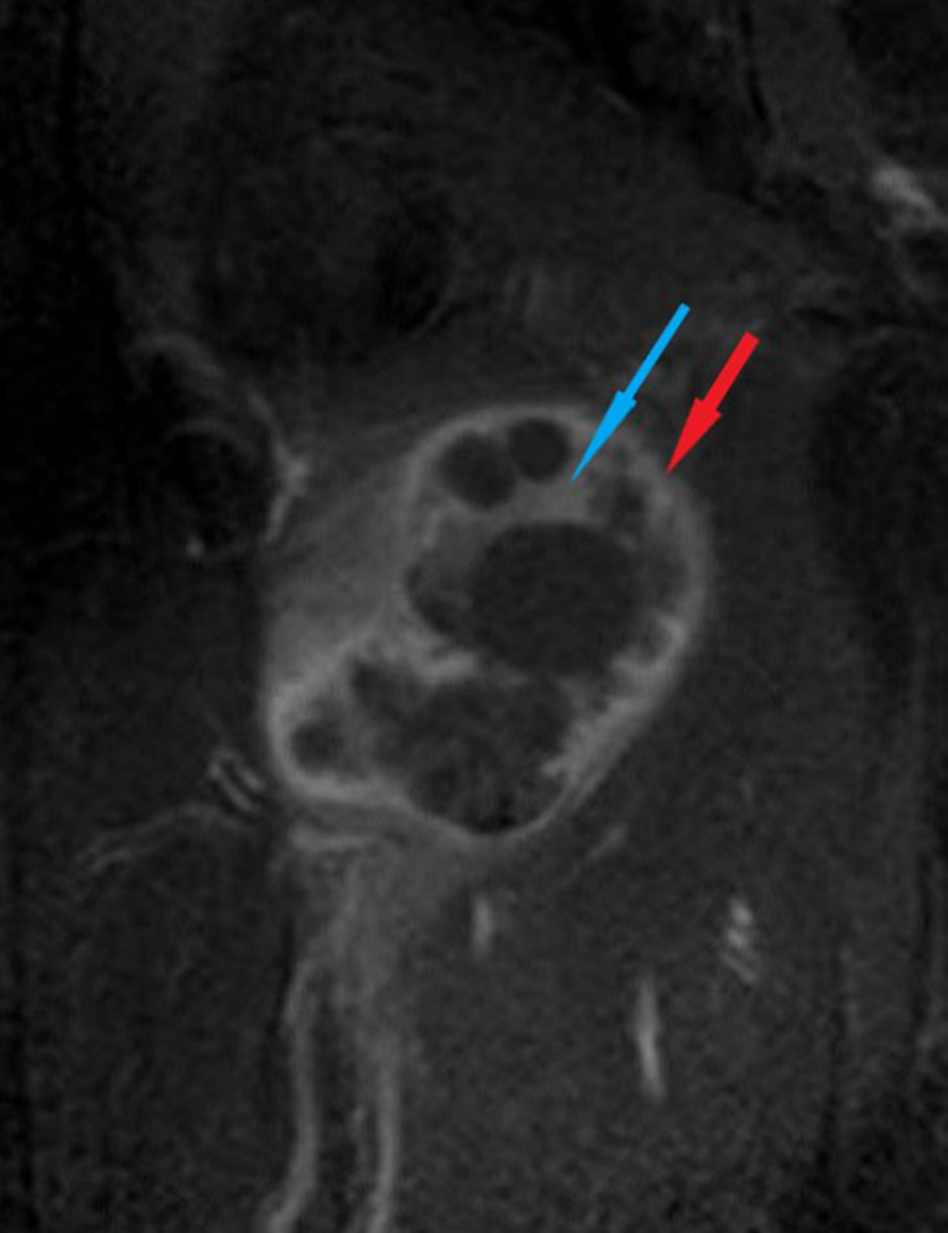
Coronal fat-suppressed *T*_1_ weighted magnetic resonance image following gadolinium administration shows a prominent peripheral enhancement (red arrow). Note that while daughter cysts do not enhance, there is an intermediate enhancement in the mother cyst (blue arrow).

Ultrasonography revealed a complex cystic lesion with multiple internal vesicles within the adductor muscle group. MRI showed that the cyst was limited in the adductor magnus muscle and was 55 × 40 × 76 mm (T x AP x CC) (Figure 1). On *T*_1_ weighted images, the daughter cysts were hypointense and the mother cyst was iso-hyperintense as compared with the adjacent muscle tissue. Both the mother and daughter cysts showed high signal intensity on *T* weighted images. And *T*_2_ weighted images nicely delineated the hypointense ectocyst and hyperintense pericyst surrounding the lesion (Figure 2). The daughter cysts were hyperintense on both diffusion-weighted images and apparent diffusion co-efficient mapping. However, prominent restriction of diffusion was recorded in the remaining parts of the mother cyst (Figure 3). Following gadolinium administration, prominent peripheral enhancement as well as intermediate enhancement of the mother cyst was observed (Figure 4). Comprehensive imaging studies revealed no other cystic lesions anywhere in the body and the patient was diagnosed with primary hydatid cyst (HC) in the adductor magnus muscle.

## Treatment

**Figure 5.**
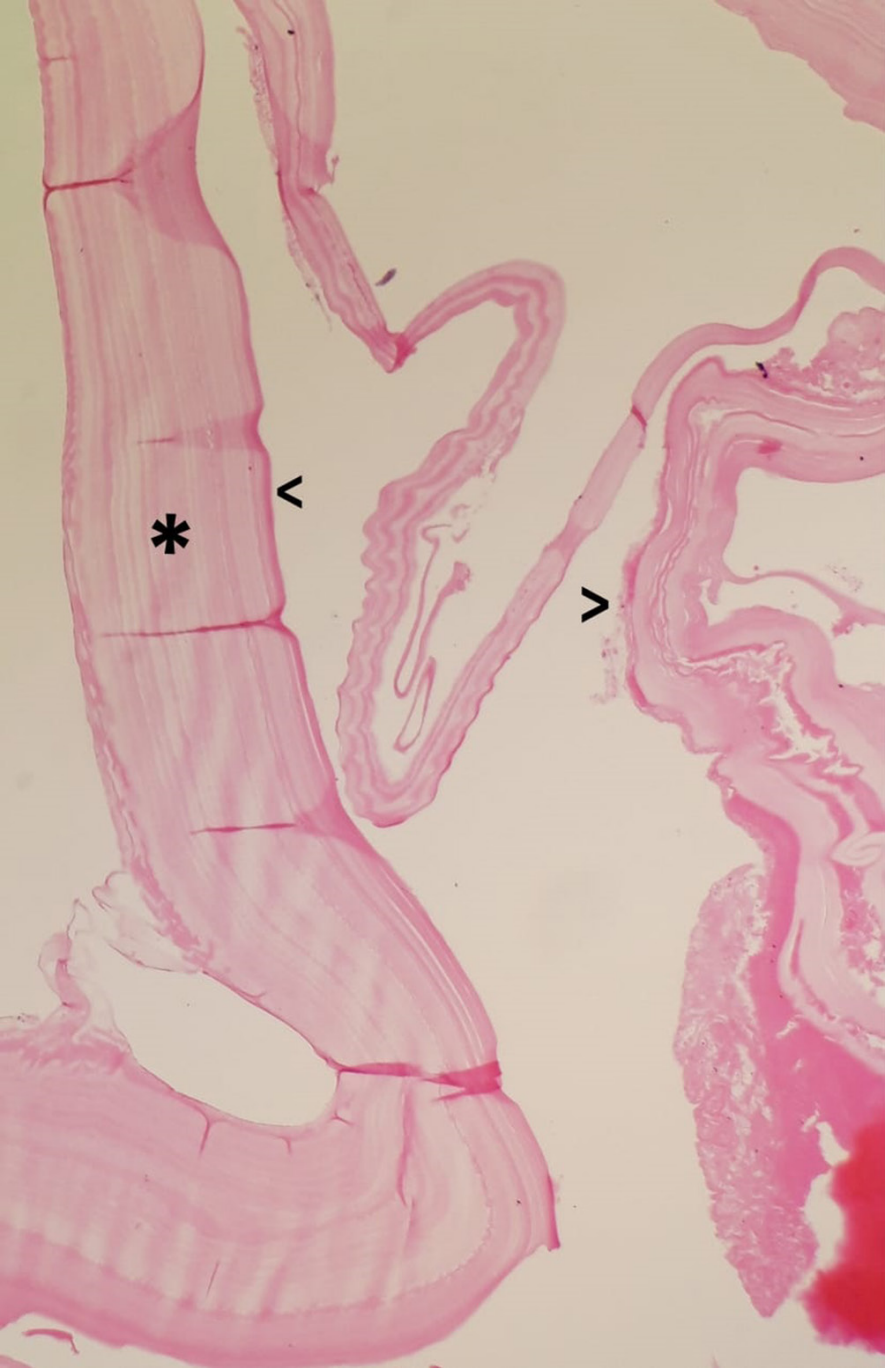
Hydatid cyst. The cyst wall; inner germinal layer (v) and laminated membrane (*). (H&E stain, (x40).

After 5 days of anthelmintic treatment with albendazole, the cyst was surgically removed. No complications developed. In the pathological examination of the removed surgical material, internal germinal layers along with laminated membranes, which are diagnostic for HC, were found (Figure 5). A 6 month albendazole treatment (with monitoring of liver function) was planned and the patient was discharged.

## Discussion

Human HC is a zoonotic disease caused by the larvae of the *Echinococcus granulosus* acquired through contact with carnivores, most commonly with dogs. Sheep are the most frequent intermediate hosts, and therefore *Echinococcus granulosus* is prevalent in the Mediterranean, Africa, South America, Middle East, Australia and New Zealand, the world's largest grazing areas.^1^ Although HC can cause disease almost anywhere in the human body, it most commonly affects the liver (65–75%) and lungs (25–30%). It rarely involves other sites such as the intra-abdominal structures, brain, heart, and musculoskeletal system (15%).^2–4^

Contractility and high lactic acid content make the muscles unfavorable for HC.^4^ Therefore, muscular HC is usually a secondary occurrence that develops as a result of migration of the larvae from the primary site after spontaneous or traumatic cyst rupture, rather than being a primary involvement. Accounting for less than 1% of all cases of hydatid disease, primary muscular hydatid disease is exceptionally rare even in endemic regions. According to the previous literature, the most frequently encountered muscles affected by the disease are the paravertebral, gluteal, and the lower extremity muscles.^3–5^ To the best of our knowledge, there are only a few case reports of primary hydatid involvement of the adductor magnus muscle in the current literature.^6–9^

Musculoskeletal hydatid disease shows no pathognomonic clinical signs and symptoms.^4^ And the contribution of serology to the diagnosis is negligible due to the high rate of false-negative results. Therefore, radiological imaging studies have a critical role in the diagnosis of the disease.^10^ Ultrasonography, CT and MRI are imaging methods that can be used in both diagnosis and selection of the appropriate treatment approach. On ultrasonography imaging, a multivesicular cyst is diagnostic for the disease. However, HC can sometimes display different appearances such as a simple cyst, a cyst with floating membranes, or a complex cystic lesion. In case, there is abundant debris and/or inflammatory material inside the cyst, it may appear as a heterogeneous solid mass mimicking a tumor on ultrasonography examination.^3–5^ CT, another imaging tool, is not a preferred method for evaluating the cyst matrix, but it is accepted as the reference method for the examination of cysts showing calcification.^10^ MRI is a perfect method to evaluate the internal structure of the HC. In addition, it provides an excellent definition of surrounding musculoskeletal structures as well as the cyst borders, thanks to its multiplanar imaging capability.^4,10^

The rim surrounding the HCs, which was originally described for hepatic and pulmonary lesions, is reported to be visible in 28–72% of cases of musculoskeletal HCs.^11^ Our case exhibited this characteristic rim which is made up of two layers: an inner hypointense layer representing the non-vascularized membrane belonging to the parasite (ectocyst) and an outer hyperintense layer representing the vascularized membrane formed in response to the host (pericyst) (Figure 2). The vascularized pericyst enhances in contrast-enhanced series.^3–5,11^ In our case, in addition to the apparent contrast enhancement in the outer membrane, a moderate enhancement was noted in the matrix of the mother cyst (Figure 4).

There is inconsistency between the previous reports on the behaviour of HCs on diffusion-weighted imaging. Inan et al reported that the apparentdiffusion coefficient (ADC) values of the HCs are significantly lower than those of simple cysts, and they commented that diffusion-weighted imaging might help in the differential diagnosis of hydatid and simple cysts.^12^ However, Oruç et al found no significant difference between ADC values of the simple cysts and those of Types 1 and 3 HCs. They reported that Type 4 HCs display significantly lower ADC values as compared with simple cysts and Types 1 and 3 HCs.^13^ The cyst we currently present showed a multivesicular configuration that fits the Type 3 HCs. And in contrast with the findings of Oruç et al, it exhibited a prominent restriction of diffusion in the mother cyst (Figure 3). On the other hand, it should be taken into consideration that, both the mentioned studies were carried out on HCs of the liver, not on those of the musculoskeletal system. Furthermore, the classification system used by Oruç et al was the Gharbi’s classification system established for the evaluation of liver HCs, not musculoskeletal HCs. The molecular characteristics of a muscular HC may differ from that of a liver HC. Therefore, it may be misleading to discuss the diffusion functions of muscle HCs by comparing them with the findings of the studies carried out on liver lesions. The diffusion restriction that we recorded in areas between daughter cysts may have occured due to the increased cellularity that developed as a result of active inflammation within the mother cyst. Actually, there is a paucity of data in the current literature concerning this topic. As a result, further case reports and comparative studies on diffusion characteristics of musculoskeletal HCs are needed to make precise comments on the diffusion-weighted imaging features of these lesions.

Treatment of muscle HDs is surgical removal with anthelmintic chemotherapy. Anthelmintic chemotherapy has been reported to reduce the rate of viable cysts within the lesion, thereby preventing relapse. It is important to remove the HC lesion as a whole without disintegration to prevent the infection from spreading to healthy tissue. It is best to remove the lesion using the same technique as the excision of a malignant tumor. An important issue to keep in mind when approaching any lesion with HC in the differential diagnosis list is that incisional biopsy and marginal excision are contraindicated in these cases. The liquid content of HCs contains a significant amount of toxic proteinous material, which is extremely risky for the host.^4,11^

## Consent for publication

Written informed consent was obtained from the patient for publication of this case report, including accompanying images.

## Learning points

1. Although very rare, adductor magnus muscle can be the primary involvement site of HC. So, HC should be included in the differential diagnosis in cases where a well-defined soft tissue mass is detected in this localization.
2. MRI is the method of choice in evaluating the internal matrix and extension of musculoskeletal HCs.
3. The rim sign on *T*_2_ weighted magnetic resonance images is a useful finding for the differential diagnosis of musculoskeletal HCs.
4. Before making definitive comments about the diffusion-weighted imaging features of musculoskeletal HCs, further case reports and comparative studies on the diffusion properties of the musculoskeletal system HCs are needed.
